# Acute effect of technique modification training on 180° change of direction performance and kinematics in adolescent male soccer players

**DOI:** 10.3389/fspor.2025.1453859

**Published:** 2025-02-11

**Authors:** Hayato Nakamura, Daichi Yamashita, Daichi Nishiumi, Naoto Nakaichi, Norikazu Hirose

**Affiliations:** ^1^ Athletic Training Laboratory, Graduate School of Sports Sciences, Waseda University, Saitama, Japan; ^2^ Japan Institute of Sports Sciences, Japan High Performance Sport Center, Tokyo, Japan; ^3^ Athletic Training Laboratory, Faculty of Sport Sciences, Waseda University, Saitama, Japan

**Keywords:** intervention, agility, turning, deceleration, acceleration, motion analysis, Pro-Agility

## Abstract

**Introduction:**

Change of direction (COD) maneuvers are frequently performed during soccer games and are critical for performance. Adolescent players often display immature COD maneuvers, suggesting that COD technique modification training may be a more effective approach for adolescent athletes. This study investigated the acute effects of COD modification training on COD performance and kinematics in male adolescent soccer players.

**Methods:**

Twenty-nine male junior high school soccer players participated in this study and were divided into two groups: 16 players underwent 15-min COD technique modification training (COD group), while 13 players engaged in 15-min linear sprint training. The participants performed Pro-Agility and 20-m sprint tests before and after the intervention, and the total times were measured. COD deficit (CODD) was calculated as the difference between these times. Center of mass (COM) velocity and trunk and lower limb kinematics were computed from three-dimensional kinematic data collected during the Pro-Agility test using a markerless motion capture system. Each section of the Pro-Agility test was divided into acceleration and deceleration phases based on the COM velocity; Stop marks the moment of direction change. Two-way (group and time) mixed ANOVA was conducted with Bonferroni corrections for *post-hoc* comparisons.

**Results:**

No significant interactions were observed in the total time of the Pro-Agility test, CODD, 20-m sprint time, or average acceleration and deceleration in each phase (*p* > 0.05). On the other hand, the COD group showed significant interactions and improvements in average deceleration from final foot (FF) contact to Stop (*p* = 0.012, *g* = 0.639), penultimate foot hip flexion angle at Stop (*p* = 0.042, *g* = 0.496), COM-FF horizontal distance at Stop (*p* = 0.008, *g* = 0.650), and FF ground contact time (*p* < 0.001, *g* = 0.803).

**Conclusion:**

A 15-min COD technique modification training led to partial, immediate improvements in kinematic parameters among adolescent soccer players but did not enhance overall COD performance or acceleration/deceleration ability.

## 1 Introduction

Change of direction (COD) maneuvers are often performed during soccer. Soccer players perform approximately 100 COD maneuvers in the 90–180° range per match (1), requiring substantial deceleration during COD. These maneuvers are common in situations involving opponents (2) and are essential for gaining temporal and spatial advantages. Consequently, improving COD performance is one of the primary goals of performance coaches who implement targeted training interventions. COD performance is typically assessed by the total time required to complete a COD task (CODTT). However, for a more detailed analysis, COD maneuvers can be broken down into four components: initial acceleration, deceleration, turning, and reacceleration (3). Previous studies have shown that deceleration before and after turning affects CODTT (4, 5), and elite soccer players complete COD tasks faster than their non-elite counterparts (6, 7).

Moreover, because CODTT is greatly influenced by linear sprint ability (8, 9), the concept of COD Deficit (CODD), calculated as the difference between the CODTT and linear sprint time over the same distance, has been proposed (8). CODD reflects time loss owing to deceleration, direction change, and reacceleration relative to linear sprinting ability, independent of linear sprint ability (8, 10). Harper et al. further divided the deceleration phase during the deceleration test into early and late halves (11). They found that a large deceleration was associated with superior physical fitness (11). Another study used a three-dimensional (3D) motion capture system to evaluate the center of mass (COM) velocity-time curve during the 505 Test, revealing significantly larger deceleration from the penultimate foot (PF) in the faster group than in the slower group (4). Therefore, examining the training effect on acceleration and deceleration in COD maneuvers in detail is essential.

Various training methods, including resistance and plyometric training, are commonly employed to improve COD. A meta-analysis by Chaabene et al. suggested that improvements from resistance training may stem from neural factors, such as increased motor unit recruitment and synchronization, as well as morphological factors, including increased muscle fiber size (12). Similarly, a meta-analysis by Asadi et al. indicates that plyometric training enhances COD performance through neuromuscular adaptations, improves motor unit recruitment, and increases eccentric strength in thigh muscles (13).

Moreover, COD techniques that optimize horizontal force orientation are essential for COD performance (4, 14). For example, a lower COM achieved through PF triple flexion and trunk inclination toward the intended direction increases stability and facilitates a more horizontal application of the braking force (4). In the turning phase, a wider final foot (FF) contact position relative to the COM plays a key role in applying propulsive force (15, 16). Dos'Santos et al. indicated that the CODTT of the 505 Test was significantly correlated with PF hip flexion angle, PF knee flexion angle, and the vector of propulsive force in FF (4). To modify such techniques, previous studies have investigated the impact of COD technique modification training on these aspects (17–19). This method involves implementing drills that incorporate various COD maneuvers, teaching technical points, and providing live feedback. A previous study reported that a 6-week COD technique modification training program in adult populations improved the CODTT of the 505 Test, as well as the PF maximum hip flexion angle and FF contact time (18). Furthermore, such technical improvements can potentially decrease the load on the FF knee, thereby mitigating the risk of severe non-contact knee injuries (17, 19, 20). For example, the increase in PF hip maximum flexion angle promoted deceleration with PF, which is associated with reducing FF knee abduction moment (17). Similarly, trunk inclination toward the intended direction, a characteristic observed in athletes with faster CODTT (4), has also been associated with a reduced risk of knee injuries (17, 20). This indicates that COD technique modification training may be effective for both performance enhancement and, in some aspects, injury prevention (17–19).

COD ability is trainable across various adolescent-age players (21, 22), who tend to exhibit immature COD maneuvers (23). Therefore, COD technique modification training may be a more effective approach for improving the COD ability in these athletes, potentially leading to immediate positive changes. Such acute changes in COD maneuvers can lead to long-term performance enhancement and injury prevention. When adolescent athletes experience injuries, their training and match participation are often limited, negatively impacting future performance and restricting their athletic development (24). Therefore, more research on training interventions for skill learning and adaptation in COD performance is needed for adolescent athletes (25).

Consequently, this study investigated the acute effects of COD technique modification training on COD performance and kinematics in male adolescent soccer players. We hypothesized that COD technique modification training would acutely improve the kinematics of COD maneuvers, enhancing deceleration ability and overall COD performance.

## 2 Materials and methods

### 2.1 Research design

This randomized controlled trial employed a pre-test/post-test design to investigate the acute effects of COD technique modification training on COD performance and kinematics in adolescent male soccer players. This study measured the 20-m sprint time, CODTT, and kinematic data in the Pro-Agility test and calculated the CODD before and after the intervention. Participants were categorized into two groups, each undergoing a different training program between the pre- and post-test. The COD technique modification training group (CODG) underwent COD technique modification training focused on refining basic COD skills (Table 1), whereas the control group (CONG) engaged in linear sprint training, excluding COD (Table 2). The training duration (15 min) and running distance (190 m) were matched between CODG and CONG to ensure comparability. 3D motion analysis was conducted using a markerless motion capture system to explore joint kinematics and the COM velocity-time curve during the Pro-Agility test to examine the effectiveness of the intervention.

**Table 1:** Fifteen-minute intervention program of COD modification training.

	Exercises	Intensity (perceived speed)	Total distance (m)	Number of decelerations and CODs	Repetitions	COD emphasis
1	5 m running—Stop—10 m running—Stop	80%	15	2	2	- Decelerate at PF
2	5 m running—90° COD—5 m sidestep—Stop	80%	10	2	2 (1 each side)	- Decelerate at PF - Push the body in the next direction at FF
3	5 m sidestep ×2–5 m running	100%	15	2	2 (1 each side)	- Decelerate at PF and drop COM - Push the body in the next direction at FF - Incline the trunk toward the next direction
4	5 m running—90° COD—5 m running—135° COD—5 m running—Stop	80% and 100%	15	3	4 (1 each side and intensity)	- Decelerate at PF - Push the body in the next direction at FF - Incline the trunk toward the next direction
5	5 m shuttle run (including 2 180° COD)	100%	15	2	2 (1 each side)	- Decelerate at PF and drop COM - Push the body in the next direction at FF - Incline the trunk toward the next direction
6	Pro-Agility run	100%	20	2	1	- Decelerate at PF and drop COM - Push the body in the next direction at FF - Incline the trunk toward the next direction
	Total		190	30		

COD, change of direction; PF, penultimate foot; FF, final foot; COM, center of mass.

**Table 2:** Fifteen-minute intervention program of linear sprint training.

Exercises	Intensity	Total distance (m)	Number of decelerations and CODs	Repetitions
10 m running	80%	10	0	2
15 m running	80%	15	0	4
15 m running	Gradually increase to maximum speed	15	0	2
15 m running	100%	15	0	4
20 m running	100%	20	0	1
Total		190	0	

### 2.2 Participants

A minimum sample size of 12 per group was determined based on a prior power analysis using G*Power (version 3.1; University of Dusseldorf, Germany), assuming an effect size of 0.33, indicating a power of 0.8 and a type 1 error (alpha level) of 0.05 (26). Consequently, 32 junior high school male soccer players from a regional-level soccer team participated in this study, with 16 participants randomly assigned to either the CODG or the CONG. However, due to scheduling conflicts on the day of the study, three participants from the CONG dropped out. Therefore, 16 players were recruited for the CODG [mean ± standard deviation (SD); age: 14.0 ± 0.6 years, height: 162.8 ± 8.6 cm, and body mass: 51.9 ± 6.1 kg], and the other 13 players acted as the CONG (age: 14.1 ± 0.7 years, height: 165.8 ± 5.5 cm, and body mass: 55.2 ± 6.4 kg). No significant differences in age, height, or body mass were observed between the groups (*p* = 0.190–0.765). This study was approved by the Ethics Review Committee on Research with Human Subjects of Waseda University (approval no. 2022-308), and all participants and their parents were informed of the benefits and risks of the investigation before signing the consent forms. None of the participants had lower limb injuries that could affect measurements or training. They had no experience with systematic physical training but regularly participated in soccer, completing four 90-min training sessions or playing one competitive match per week.

### 2.3 Procedure

All measurements were completed on the same day between 18:00 and 21:00. Participants underwent anthropometric measurements wearing shorts, a T-shirt, and socks. After a standardized 10-min warm-up consisting of forward and backward jogging, sidestepping, dynamic stretching, and sprints, participants performed the pre-tests, including the 20-m sprint and Pro-Agility tests in a random order, with times measured using a timing gate system. A rest period of at least 2 min was allowed between trials or measurements to minimize fatigue. Then, participants completed a 15-min intervention program, and after 4 min of passive recovery, they performed the 20-m sprint and Pro-Agility tests in a random order as the post-tests. These tests and training sessions were conducted on a synthetic turf pitch while wearing soccer shoes.

#### 2.3.1 Anthropometric measurements

Height was measured to the nearest 0.1 cm using a portable stadiometer (Seca 213, Hamburg, Germany). Body mass was measured to the nearest 0.1 kg using a body composition analyzer (HD-660, TANITA, Japan). Leg length was measured twice, from the greater trochanter to the lateral malleolus, using a measuring tape while the participant stood. The average values for both legs were used in the analysis. This measurement showed high intra-rater reliability, with an intraclass correlation coefficient (ICC_2,2_) of 0.998–0.999.

#### 2.3.2 20-m sprint

The 20-m sprint time was measured using a timing gate system (VoltOnoSprint; S-CADE, Tokyo, Japan), positioned at the start and goal lines and set to a height of approximately 0.8 m. Participants adopted a two-point stance 0.5 m behind the start line and started with the examiner's verbal signal. They were instructed to start without countermovement and sprint as quickly as possible. The test was performed twice during the pretest, and the average time was calculated. The post-test was performed only once.

#### 2.3.3 Pro-Agility test

A Pro-Agility test was performed in three trials after sufficient familiarization. Participants adopted a two-point stance 0.5 m behind the start line and started with the examiner's verbal signal. They were instructed to perform the test as quickly as possible, using their dominant (kicking) leg to turn, ensuring they either touched or crossed the line at each turn. The CODTT was measured using a timing gate system (VoltOnoSprint, S-CADE, Tokyo, Japan) positioned at the start and goal line and set at approximately 0.8 m. 3D kinematic data were obtained using a markerless motion capture system (Theia3D, v2023, Theia Markerless, Kingston, ON, Canada) and 13 cameras (Sony RX0 II, Sony Corporation, Tokyo, Japan) at 120 fps. Trials were repeated for premature direction changes or slips after rest until three valid attempts were recorded, and the average value of the three trials was adopted for analysis.

### 2.4 Data analysis

#### 2.4.1 CODD

CODD was calculated by subtracting the 20-m sprint time from the Pro-Agility CODTT (CODTT—20-m sprint time).

#### 2.4.2 3D kinematic data

Video data were processed using the Theia3D software to obtain 3D pose estimates of the participants using the default inverse kinematics model. The 3D pose estimates for each body segment were exported for further analysis using Visual3D (x64 Professional v2023.03.1; C-Motion Inc., Germantown, MD) (27). Kinematic variables were smoothed using generalized cross-validation spline smoothing with a 10 Hz cut-off frequency. Lower limb joint angles were calculated using the standard Cardan rotation sequence (X-Y-Z) equivalent to the joint coordinate system (28). The trunk inclination angle was defined as the angle relative to the global coordinate system.

**Figure 1:**
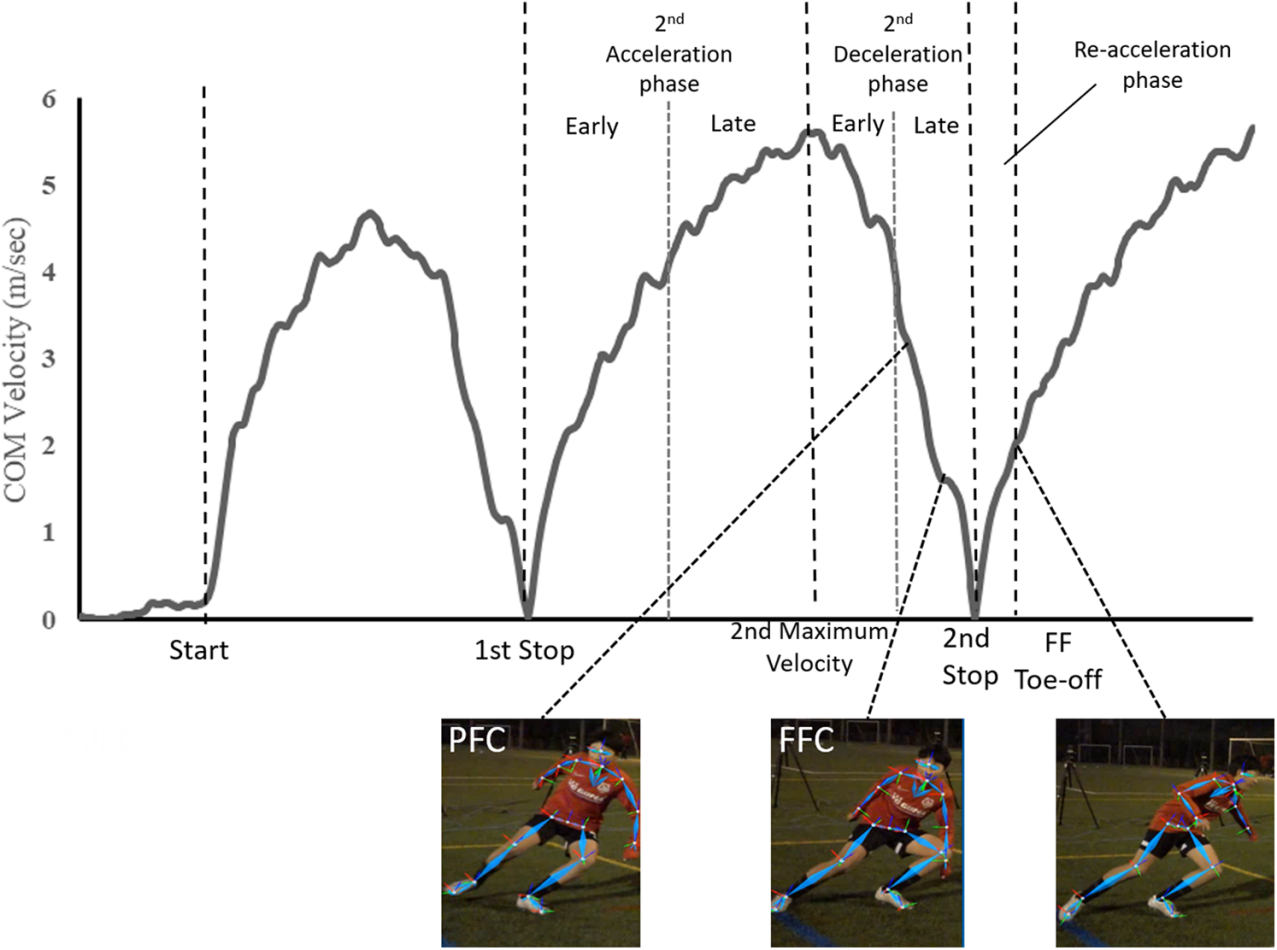
Phase division of the Pro-Agility test based on COM velocity.

In this study, we focused on the 10-m interval and the subsequent reacceleration phase, as it is the most demanding part of the Pro-Agility test (10). The phases were defined from the velocity-time curve of the COM (Figure 1). The point at which the COM velocity approached closest to zero was defined as the “Stop.” The 10-m interval was designated as the acceleration and deceleration phases, and each phase was further divided into two sub-phases of equal duration, defined as the “Early” and “Late.” Additionally, the phase from the 2nd Stop to the toe-off of the FF was defined as the reacceleration phase.

To evaluate the rate of acceleration and deceleration, we calculated the average acceleration (Acc) and deceleration (Dec) for each phase. Additionally, we specifically calculated the Dec for PF and FF contact (PFC and FFC, respectively) events during the deceleration phase (i.e., PFC-FFC, FFC-Stop, and PFC-Stop). Foot contact was defined as the first occurrence of a peak in either heel or toe vertical acceleration (29). Toe-off was defined as the toe peak vertical jerk, which is the first derivative of acceleration with respect to time (29). To evaluate the trunk and lower body kinematics during deceleration, the PF hip and knee flexion angles, trunk inclination angle, COM height, and horizontal separation distance between the COM and FF (the midpoint of the toe and heel) were calculated. The COM height was normalized to the individual leg length.

### 2.5 Intervention program

After the pretest, participants underwent approximately 15 min of training (either COD modification training or linear sprint training). Each training session was classified into two groups of approximately eight participants, led by the principal researcher, a physical fitness coach certified by the Japan Football Association.

The COD technique modification training included drills at various angles, distances, efforts, and focuses based on previous studies (18, 19) (Table 1). The training focused on three technical aspects based on previous studies: (1) “Decelerate at the penultimate foot and drop COM” (to increase deceleration in PF and PF hip and knee flexion and to the lower COM); (2) “Push the body in the next direction at FF” (to increase horizontal propulsive force, resulting in getting larger COM velocity for next direction); (3) “Incline the trunk toward the next direction” (to align COM in a more effective position to apply the braking and propulsive force horizontally and minimize COM displacement relative to the turning line). Participants were provided with verbal coaching on the aforementioned points before the drill to ensure they understood the key aspects of the upcoming drill. During the drills, coaching on these points was performed using short phrases at appropriate times. After each trial, participants received verbal feedback specifying the aspects that were performed well and the areas needing improvement concerning the aforementioned points.

The CONG conducted linear sprint training without COD maneuvers to eliminate differences caused by increased muscle temperature and post-activation potentiation. The training was designed to match the duration and total distance of the COD technique modification training (Table 2). No special instructions were given to the CONG, except for explaining the distance and intensity of the exercises.

### 2.6 Statistical analysis

All statistical analyses were performed using SPSS software (v28, SPSS, Inc., Chicago, IL, USA). The normality of all variables was assessed using the Shapiro–Wilk test. The reliability of the kinematic data from the Pro-Agility test was assessed using ICC_2,*k*_, and the coefficient of variation (CV) was calculated using data from all 29 participants. Minimum acceptable reliability was determined with an ICC ≧ 0.7 and CV ≦ 15%, and variables demonstrating low reliability were removed from further analysis (4).

A two-way mixed analysis of variance (ANOVA), with a between-subject factor (CODG and CONG) and a within-subject factor (pre- and post-intervention measures), was used to identify significant interactions (group × time) for outcomes. A Bonferroni-corrected pairwise comparison was used for further analyses when significant interactions were observed. Partial eta squared (partial *η*^2^) effect sizes were calculated for all ANOVAs and evaluated as follows: small (0.010–0.059), medium (0.060–0.149), and large (≧0.150). Pre-to-post changes in variables for each group were assessed using a paired-sample *t*-test, and the magnitudes of differences were evaluated using Hedges' *g* effect sizes, interpreted as trivial (≦0.19), small (0.20–0.59), moderate (0.60–1.19), large (1.20–1.99), very large (2.00–3.99), and extremely large (≧4.00). Statistical significance was set at *p* < 0.05 for all analyses.

## 3 Results

Almost all measurements and analysis variables demonstrated high intra-session reliability (Pre: ICC_2,*k*_ = 0.701–0.850, CV = 1.0–14.3%; Post: ICC_2,*k*_ = 0.707–0.934, CV = 1.4–13.8%) except for Early Dec, Late Acc, PF hip flexion angle at PFC, PF knee flexion angle at PFC and Stop, FF hip flexion angle at Stop, and FF knee flexion angle at FFC and Stop (Table 3). These variables showed low reliability and were excluded from further analysis.

**Table 3:** Reliability of completion times and phase- and event-specific variables.

Variables	Event	**ICC_2,k_**		CV (%)	
		Pre	Post	Pre	Post
Completion times					
CODTT	—	0.850	0.934	1.8	1.4
20 m sprint	—	0.955	—	1.0	—
Variables at 2nd turn					
Early Acc	—	0.851	0.856	4.0	4.0
Late Acc	—	0.611	0.531	10.7	11.8
Early Dec	—	0.493	0.189	22.4	24.9
Late Dec	—	0.758	0.792	6.5	6.2
Re-Acc	—	0.718	0.770	6.1	7.5
Dec	PFC-FFC	0.773	0.840	12.3	13.8
	FFC-Stop	0.701	0.734	10.7	11.4
	PFC-Stop	0.788	0.766	5.8	9.3
Contact time	FF	0.768	0.709	14.3	13.2
PF hip flexion angle	PFC	0.575	0.635	10.0	10.9
	FFC	0.831	0.797	7.9	6.7
	Stop	0.856	0.783	7.2	7.5
PF knee flexion angle	PFC	0.485	0.482	21.3	20.1
	FFC	0.744	0.750	8.5	5.2
	Stop	0.826	0.647	6.5	5.4
FF hip flexion angle	FFC	0.814	0.789	13.3	13.3
	Stop	0.831	0.778	19.7	20.0
FF knee flexion angle	FFC	0.506	0.554	16.4	14.1
	Stop	0.650	0.333	12.0	12.1
COM height	PFC	0.830	0.920	3.1	3.6
	FFC	0.910	0.928	3.4	2.9
	Stop	0.880	0.861	4.0	3.9
COM—FF horizontal distance	FFC	0.770	0.845	3.3	2.4
	Stop	0.710	0.707	6.8	6.1
Trunk inclination angle	PFC	0.771	0.790	11.6	12.2
	FFC	0.835	0.859	9.4	8.6
	Stop	0.840	0.788	9.9	11.0

CV, coefficient of variation; CODTT, total time required to complete a COD task; Acc, mean acceleration of center of mass during the acceleration phase; Dec, mean deceleration of center of mass during the deceleration phase; COM, center of mass; PF, penultimate foot; FF, final foot; PFC, penultimate foot contact; FFC, final foot contact.

Based on the two-way mixed ANOVA, no significant interaction was observed in CODTT, CODD, or 20-m sprint time. No significant interactions were observed in any of the Acc or Dec in each acceleration and deceleration phase (Table 4). A large, significant interaction for Dec in PFC-Stop was observed (*p* = 0.023, Partial *η^2^* = 0.178) without a significant increase in either group (Table 5). A medium, significant interaction for Dec in FFC-Stop was observed (*p* = 0.048, Partial *η^2^* = 0.137), with CODG showing a significant, moderate increase (*p* = 0.012, *g* = 0.639) (Table 5). A large, significant interaction for FF contact time was observed (*p* = 0.036, Partial *η^2^* = 0.152), with CODG showing significant moderate reduction (*p* < 0.001, *g* = 0.803) (Table 5).

**Table 4:** ANOVA and *post-hoc* results for COD performance and average acceleration and deceleration in each acceleration and deceleration phase.

Variables	Group	Pre	Post	Interaction		*Post-hoc*	
		Mean ± SD	Mean ± SD	*p*	Partial *η*^2^	*p*	Hedges’ *g*
CODTT (s)	COD	5.32 ± 0.36	5.32 ± 0.33	0.077	0.112	—	—
	CON	5.18 ± 0.15	5.24 ± 0.19			—	—
20-m Sprint (s)	COD	3.42 ± 0.32	3.42 ± 0.23	0.956	0.000	—	—
	CON	5.18 ± 0.15	5.24 ± 0.19			—	—
CODD (s)	COD	1.90 ± 0.14	1.89 ± 0.18	0.137	0.080	—	—
	CON	1.83 ± 0.14	1.89 ± 0.12			—	—
Early Acc (m/s^2^)	COD	5.00 ± 0.63	4.91 ± 0.56	0.990	0.000	—	—
	CON	5.10 ± 0.42	5.01 ± 0.38			—	—
Late Dec (m/s^2^)	COD	9.49 ± 1.14	9.33 ± 0.80	0.556	0.013	—	—
	CON	9.60 ± 1.17	9.25 ± 1.42			—	—
Re-Acc (m/s^2^)	COD	6.47 ± 0.75	6.33 ± 0.87	0.914	0.000	—	—
	CON	6.51 ± 0.40	6.32 ± 0.86			—	—

CODTT, total time required to complete a COD task; CODD, change of direction deficit; Acc, average acceleration of center of mass during the acceleration phase; Dec, average deceleration of center of mass during the deceleration phase.

**Table 5:** ANOVA and *post-hoc* results for kinematic variables at 2nd turn.

Variables	Event	Group	Pre	Post	Interaction		*Post-hoc*	
			Mean ± SD	Mean ± SD	*p*	Partial *η*^2^	*p*	Hedges’ *g*
Dec (m/s^2^)	PFC-FFC	COD	7.54 ± 1.35	7.33 ± 1.36	0.432	0.023	—	—
		CON	8.91 ± 2.51	8.16 ± 2.63			—	—
	FFC-Stop	COD	14.7 ± 2.36	16.3 ± 2.64	0.048*	0.137	0.012*	0.623
		CON	14.3 ± 2.57	14.0 ± 2.22			0.707	—0.121
	PFC-Stop	COD	10.7 ± 1.06	11.2 ± 1.16	0.023*	0.178	0.097	0.439
		CON	11.3 ± 1.92	10.8 ± 2.38			0.100	—0.224
Contact time (s)	FF	COD	0.32 ± 0.07	0.27 ± 0.06	0.036*	0.152	0.000**	—0.748
		CON	0.30 ± 0.08	0.29 ± 0.06			0.638	—0.137
PF hip flexion angle (°)	FFC	COD	83.2 ± 13.7	87.7 ± 10.8	0.099	0.098	—	—
		CON	86.0 ± 10.8	84.1 ± 11.0			—	—
	Stop	COD	83.7 ± 16.1	90.5 ± 9.89	0.021*	0.183	0.042*	0.496
		CON	90.4 ± 13.0	85.5 ± 15.0			0.178	—0.338
PF knee flexion angle (°)	FFC	COD	106.2 ± 16.1	106.9 ± 9.39	0.749	0.004	—	—
		CON	106.1 ± 13.4	105.4 ± 11.6			—	—
FF hip flexion angle (°)	FFC	COD	39.6 ± 10.0	39.3 ± 7.04	0.779	0.003	—	—
		CON	37.0 ± 8.54	36.0 ± 9.84			—	—
COM height (m/leg length)	PFC	COD	0.92 ± 0.05	0.92 ± 0.05	0.279	0.043	—	—
		CON	0.93 ± 0.09	0.94 ± 0.09			—	—
	FFC	COD	0.81 ± 0.05	0.79 ± 0.04	0.041*	0.145	0.307	—0.431
		CON	0.81 ± 0.09	0.83 ± 0.09			0.062	0.215
	Stop	COD	0.80 ± 0.05	0.79 ± 0.05	0.112	0.091	—	—
		CON	0.79 ± 0.10	0.81 ± 0.09			—	—
COM—FF horizontal distance (m)	FFC	COD	0.67 ± 0.05	0.68 ± 0.05	0.187	0.064	—	—
		CON	0.68 ± 0.03	0.67 ± 0.03			—	—
	Stop	COD	0.56 ± 0.06	0.60 ± 0.06	0.006**	0.248	0.008**	0.650
		CON	0.61 ± 0.06	0.59 ± 0.04			0.237	—0.380
Trunk inclination angle (°)	PFC	COD	36.4 ± 7.05	39.9 ± 8.07	0.256	0.047	—	—
		CON	35.3 ± 9.01	35.7 ± 4.60			—	—
	FFC	COD	48.2 ± 9.29	50.2 ± 10.5	0.297	0.040	—	—
		CON	47.9 ± 10.4	46.9 ± 6.82			—	—
	Stop	COD	56.8 ± 9.94	56.0 ± 9.46	0.434	0.023	—	—
		CON	57.1 ± 12.8	54.3 ± 12.8			—	—

COM, center of mass; PF, penultimate foot; FF, final foot; PFC, penultimate foot contact; FFC, final foot contact.

**p < 0.01; *p < 0.05.

A large, significant interaction for the PF hip flexion angle at Stop was observed (*p* = 0.021, Partial *η^2^* = 0.183) with CODG showing significant small increase (*p* = 0.042, *g* = 0.496) (Table 5). A medium, significant interaction for COM height at FFC was observed (*p* = 0.041, Partial *η^2^* = 0.145) without significant improvement in either group (Table 5). A large, significant interaction for COM-FF horizontal distance at Stop was observed (*p* = 0.006, Partial *η^2^* = 0.248), with CODG showing a significant, moderate increase (*p* = 0.008, *g* = 0.650) (Table 5). No significant interactions or pre-to-post changes were observed for the other kinematic variables (Table 5).

## 4 Discussion

This study aimed to investigate the acute effect of a 15-min COD technique modification training with verbal instruction on COD performance and kinematics in adolescent male soccer players. The primary findings were that, although no acute enhancement of CODTT and CODD was observed, the intervention partially but immediately improved kinematics, including a shorter FF contact time, greater Dec in FFC-Stop, a greater PF hip flexion angle at Stop, and a longer COM-FF horizontal distance at Stop. These acute kinematic improvements may be the first step toward long-term adaptation in COD performance and injury risk reduction in adolescent athletes.

CODTT, CODD, and 20-m sprint time did not change after the 15-min intervention in either group. This suggests that fatigue did not influence the results. A previous study reported that a 6-week COD technique modification training program consisting of 12 sessions improved CODTT in the 505 Test (18). While no studies examined the acute effect of training aimed at improving COD performance, another study on adult sprinters reported an acute effect of a 10-min mini-hurdle drill, which increased step frequency while maintaining step length, resulting in improved linear sprint time (30). Nevertheless, no significant improvements in CODTT or CODD were observed in this study. COD maneuvers comprising multiple elements—initial acceleration, deceleration, turning, and reacceleration (3)—require complex motor control at high speed, and adolescent players tend to exhibit immature COD maneuvers (23). Indeed, considerable variability was observed in joint kinematics at the FF during the pre-test (CV > 15%), suggesting that participants may have had immature COD techniques. Improving such complex maneuvers requires repeated closed drills with conscious effort (25). While the training session in this study emphasized decelerating with the PF, lowering the COM, and pushing the body at the FF, a single 15-min training session may not be sufficient to optimize these techniques. Additionally, it should be noted that similar to previous studies, this training incorporated not only 180° COD but also COD at various angles required in soccer (31) for long-term progression (17–19). Therefore, by repeatedly performing such training, gradual changes in kinematics can be expected, which may lead to long-term enhancement in performance.

Conversely, the average deceleration significantly increased only during the FFC to Stop. This is interesting because no direct instructions were provided for deceleration in the FF. Indeed, some kinematic variables during the FFC changed after the intervention, one of which was the reduction in FF contact time. A previous study indicated a significant correlation between an increased PF hip flexion angle and reduced FF contact time (16). The reduction in FF contact time indicates a shorter turning phase and represents one of the key components of COD performance (18, 32). Furthermore, the CODG maintained its COM further from the FF after the intervention. Extending the COM-FF horizontal distance is believed to enable a more horizontally applied ground reaction force, which is beneficial for COD performance (15, 16). Although the CODTT did not improve in this study, the training successfully achieved rapid deceleration and an acute reduction in turn duration. Therefore, these acute kinematic changes induced by the 15-min intervention may positively influence phase-specific performance.

These kinematic changes may also contribute to the prevention of severe knee injuries, which are more commonly associated with COD maneuvers than with other movements (33). High-intensity deceleration, a key component of COD, can cause tissue damage and increase the risk of injury (34), highlighting the importance of improving braking mechanics. In this study, a significant increase in the PF hip flexion angle was observed, which may enhance stability and support braking force production during PFC (3, 4), potentially reducing FF knee abduction moment indirectly without compromising performance. Although previous studies have reported that greater trunk inclination toward the intended direction during the FFC is associated with smaller knee abduction moments (35, 36), the absence of significant changes in the trunk inclination angle after the intervention leaves this relationship unclear. Nevertheless, a scoping review by Dos'Santos et al. (2019) indicates that long-term COD technique modification training is an effective strategy for reducing knee joint loading (37). Consequently, incorporating this training continuously (e.g., into warm-up routines) over an extended period may benefit subsequent athletic performance and lead to definitive improvements in injury prevention.

While the improvement in deceleration during FFC and the longer COM-FF horizontal distance at Stop were observed, no significant changes were found in subsequent acceleration phases (Re-Acc and Early Acc). Although many drills in the training program emphasized pushing the body in the next direction at the FF, this did not translate into expected outcomes. A previous study indicated that a more horizontally oriented propulsive force vector in the FF is related to enhanced exit velocity after turning (18), and one strategy to achieve this is to incline the trunk toward the next direction (4, 38). Despite emphasizing trunk inclination toward the next direction, no significant improvement was observed in the trunk inclination angle at Stop. Achieving meaningful improvements in this kinematic variable may demand an extended focus, highlighting the importance of integrating such targeted exercises into a broader training regimen.

Similarly, despite emphasizing deceleration in the PF during training, no improvement was observed in deceleration or kinematic variables from PFC to FFC. During the entire phase from FFC to Stop, the PF remained in contact with the ground, and hip joint of the PF remained flexed. This suggests that the instruction “Decelerate at the penultimate foot and drop COM” was reflected not during the PF single-limb support phase but rather in the double support phase of the FF and PF. Furthermore, to achieve greater deceleration, it is crucial to incline the trunk toward the next direction and lower the COM at PFC (3, 4), but no changes were observed in these kinematic variables. Considering the findings of Dos’Santos et al. (2021), who reported no changes in trunk angle during deceleration even after twelve sessions of technique modification training (18), implementing drills specifically focusing on trunk posture might be more effective.

This study had a few limitations. First, because the measurements and interventions were conducted in a practical setting, the kinetics of the Pro-Agility test were not measured. A previous study reported significant improvements in braking and propulsive force vectors after a 6-week COD modification training (18). Therefore, further research should investigate acute effects on the kinetics of Pro-Agility tests. Second, the 15-min training duration may have resulted in fewer repetitions, making the progression more challenging. Further research should consider extending the duration of training interventions to increase repetitions and progress the drill, and investigate the differences in acute effects. Finally, the participants were male adolescent soccer players, and it can be inferred that they included players at various stages of maturity. However, since the participants were not categorized by maturity, the investigation of acute effects is limited to overall male junior high school players. The training effects may vary depending on maturation (39); however, the present study did not compare effects based on maturity. Moreover, it remains unclear whether these findings in the present study can be generalized to athletes of different ages and sexes. Therefore, further research is needed to investigate the differences in the acute effects of COD modification training according to maturity levels, ages, and sexes.

## Data Availability

The original contributions presented in the study are included in the article/Supplementary Material, further inquiries can be directed to the corresponding author.
